# INTEGRATING HEALTH POLICY EDUCATION INTO AN UNDERGRADUATE PSYCHOLOGY COURSE

**DOI:** 10.1093/geroni/igad104.0370

**Published:** 2023-12-21

**Authors:** Kelly O’Malley, Kirsten Graham

**Affiliations:** VA Boston Healthcare System, Boston, Massachusetts, United States; Southern Utah University, Cedar City, Utah, United States

## Abstract

Education on health policy processes are lacking in psychology training programs. Psychologists can be helpful to policy making and evaluation processes; however, few have the training, experience, or exposure to health policy necessary to feel comfortable entering into this area. Health policy education was integrated into an existing undergraduate psychology adult development and aging curriculum. Students completed weekly knowledge quizzes, two health policy specific assignments, and a class lecture on the intersection of health policy, aging, and psychology. N = 97 students (Mage = 21.54, SD = 4.94) over six semesters completed pre-post knowledge, skill, and ability assessments. Paired samples t-tests indicated increased knowledge t(96)= -5.89, p < .001, Cohen’s d = .60, and understanding of health policy making t(94)= -5.69, p < .001, Cohen’s d = .58; and ability to critically evaluate health policies t(96)= -3.03, p = .003, Cohen’s d = .31. Students identified psychology t(95)= -5.20, p < .01, Cohen’s d = .53 and psychological research t(95)= -3.71, p < .001, Cohen’s d = .38 as having a greater role in health policy following the course. Students felt they would use the health policy skills and knowledge learned in this course and agreed the policy related topics and assignments were valuable. Integrating health policy education into an undergraduate psychology course was feasible and had a positive impact on student’s knowledge, skills, and abilities in this domain. This format may be appropriate for graduate psychology training and may help build psychologists confidence to participate in health policy activities.

